# Exploratory profiling of serum small extracellular vesicle-associated miRNAs as candidate biomarkers for Moyamoya disease

**DOI:** 10.1016/j.bbrep.2026.102686

**Published:** 2026-07-20

**Authors:** Lin Yan, Hao Ding, Ruifang Zhao, Hanqing Chen

**Affiliations:** a Department of Neurosurgery, Xuanwu Hospital, Capital Medical University, Beijing, 100053, China; b China International Neuroscience Institute (China-INI), Beijing, 100053, China; c Beijing Key Laboratory for Drug Delivery Nanocarriers, CAS Center for Excellence in Nanoscience, National Center for Nanoscience and Technology, Beijing, 100190, China; d Beijing Key Laboratory of Environment and Aging, Department of Nutrition & Food Hygiene, School of Public Health, Capital Medical University, Beijing, 100069, China

**Keywords:** Moyamoya disease, Extracellular vesicles, MicroRNAs/miRNAs, Biomarkers, Weighted gene co-expression network analysis (WGCNA)

## Abstract

**Purpose:**

Moyamoya disease (MMD) is a chronic progressive cerebrovascular disease with elevated rates of mortality and disability in children and adults. Therefore, early and accurate identification of MMD is crucial for good prognosis, effective therapeutics, and increased clinical survival rates. Serum small extracellular vesicles (EVs) have attracted significant attention as innovative liquid biopsy biomarkers for screening and diagnosing MMD.

**Patients and methods:**

We performed small RNA (sRNA) sequencing to determine the miRNA profiles of serum-derived EVs and integrated the Weighted gene co-expression network analysis to identify the serum exosomal miRNA biomarkers within a screening cohort comprised of 10 adult MMD patients (38.7 ± 12.2 years), along with 10 age and gender-matched healthy controls (adult HCs, 41.0 ± 15.0 years) and 11 pediatric MMD patients (8.6 ± 2.2 years).

**Results:**

Our results indicated that mRNA-derived fragments represented the largest annotated mapped fraction, whereas miRNAs were the principal small regulatory RNA class further analyzed for candidate biomarker discovery. Among them, specific EV-derived miRNAs, miR-378c, miR-205-5p and miR-374a-5p, were identified as closely related to disease progression in adult MMD patients. This miRNA exhibited significantly altered expression levels in adult MMD patients but not in pediatric cases, suggesting a potential role in the pathophysiology of MMD that might be associated with age-dependent factors or stages of disease development. The findings of this study emphasize the potential application of serum-EV-derived miRNAs as biomarkers for MMD, which could serve as an indicator for disease progression in adult patients. While the profiles of pediatric MMD were less significantly altered, the association of EV-derived miRNAs with adult disease progression presents a promising lead for adult-specific biomarkers.

**Conclusion:**

These results provide a foundation for larger-scale studies with longitudinal follow-up to validate using serum exosomal miRNAs, particularly miR-205-5p and miR-374a-5p as non-invasive diagnostic and prognostic biomarkers for patients with adult MMD, enhancing clinical management and patient outcomes.

## Highlights

• Exosomal miRNA signatures distinguish Moyamoya disease patients.
• MiR-205-5p and miR-374a-5p act as central ceRNA hubs in MMD.
• A random forest model achieves AUC 0.87 for noninvasive MMD diagnosis.
• WGCNA links MMD-associated miRNAs to mitochondrial dysfunction.
• Integrative liquid biopsy enables early detection and personalized MMD management.

## 1 Introduction

Moyamoya disease (MMD) is a chronic, progressive cerebrovascular disorder characterized by bilateral stenosis or occlusion of the terminal internal carotid arteries and proximal anterior and middle cerebral arteries [1,2]. Patients face high risks of ischemic and hemorrhagic stroke due to the formation of fragile collateral networks [3]. The incidence and prevalence of MMD are 0.59 and 1.01 per 100,000 person-years in China [4], respectively. With a notably high prevalence in East Asian populations, MMD is clearly important in pediatric cerebrovascular disease, but adult stroke has many more common etiologies [5,6]. The annual stroke rates after indirect revascularization procedures are 0%-2.3% and 0%-14.3% in pediatric and adult patients with MMD [5,6]. The clinical course is often severe, marked by recurrent ischemic events, hemorrhagic strokes, and progressive cognitive decline, imposing a substantial burden on patients and healthcare systems [7]. Timely surgical intervention is an effective treatment for reducing the long-term risk of new-onset stroke in MMD patients [8]. Therefore, this clinical imperative has fueled the search for reliable non-invasive diagnostic methods.

The definitive diagnosis of MMD currently relies on invasive digital subtraction angiography (DSA), which remains the gold standard for visualizing the characteristic steno-occlusive changes and moyamoya collateral networks[9]. Magnetic resonance imaging and angiography (MRI/MRA) provide non-invasive alternatives but lack the sensitivity and specificity of DSA for definitive diagnosis, particularly in early-stage disease [10,11]. The diagnostic paradigm presents significant clinical challenges, including the invasive nature of DSA, which carries procedural risks, limits its suitability for repeated monitoring of disease progression or postoperative surveillance, and hinders large-scale screening in at-risk populations [12,13]. Furthermore, the diagnosis is often delayed until symptomatic stroke occurs, missing a critical window for potential early intervention [12,13]. Surgical revascularization, including direct and indirect bypass procedures, is the primary treatment to restore cerebral perfusion and prevent future strokes[14]. However, the timing of surgery and the assessment of its efficacy are complicated by the lack of reliable, dynamic [14], and non-invasive biomarkers that reflect the underlying molecular pathology and disease activity.

In recent years, liquid biopsy has emerged as a potential approach in oncology and neurology, offering a minimally invasive window into disease biology through the analysis of circulating biomarkers [8]. Among these, extracellular vesicles, particularly small extracellular vesicles (EVs) (30-150 nm in diameter), have garnered immense interest [15]. EVs are lipid bilayer-enclosed nanovesicles actively secreted by virtually all cell types, including endothelial cells, neurons, and glial cells within the neurovascular unit [[16], [17], [18]]. They carry a rich molecular cargo, proteins, lipids, and nucleic acids (including miRNAs, mRNAs, and lncRNAs), that mirrors the physiological and pathological state of their parent cells [[19], [20], [21]]. Critically, their lipid bilayer confers remarkable stability to their contents against enzymatic degradation in the circulation, making them a superior candidate carrier for biomarker discovery [20]. MicroRNAs (miRNAs) are small (∼22 nt), non-coding RNAs that function as key post-transcriptional regulators of gene expression by binding to complementary sequences on target mRNAs, leading to translational repression or mRNA degradation [[22], [23], [24]]. Dysregulated miRNA expression profiles have been implicated in the pathogenesis of numerous cerebrovascular and neurodegenerative diseases, including atherosclerosis, stroke, and Alzheimer's disease [25,26]. Serum small EVs are derived from multiple cell types, including endothelial cells, immune cells, platelets, erythrocytes, and other peripheral tissues. Therefore, serum small EV-associated miRNAs should be interpreted as systemic circulating biomarkers rather than tissue-specific readouts of intracranial vascular pathology. [27]. While recent pioneering studies have begun to explore exosomal cargo in MMD [15,27], suggesting roles in endothelial dysfunction and angiogenesis, a comprehensive, unbiased profiling of serum exosomal miRNAs and their integrated diagnostic utility remains largely unexplored.

To address this gap, we hypothesized that serum-derived EVs from MMD patients harbor distinct miRNA fingerprints mechanistically linked to disease pathogenesis and of significant diagnostic value. In this study, we performed deep small RNA sequencing on serum EVs from a well-characterized cohort of adult and pediatric MMD patients and matched healthy controls. Using an integrative framework combining differential expression analysis, weighted gene co-expression network analysis (WGCNA), and competing endogenous RNA (ceRNA) network construction, we identified disease-associated miRNA modules and regulatory hubs. Finally, we developed and validated a machine learning-based diagnostic model to translate these molecular signatures into a candidate biomarker for MMD.

## 2 Experimental section

### 2.1 Study design and ethical approval

This study was conducted in accordance with the World Medical Association Declaration of Helsinki (Ethical Principles for Medical Research Involving Human Subjects) and was approved by the Ethical Review Committee of Xuannwu Hospital of Capital Medical University (2023[051]). All procedures were conducted in accordance with the ethical standards of the Ethical Guidelines of Xuanwu Hospital, which align with the Declaration of Helsinki of 1975 and its 1983 revision. Written informed consent was obtained from all adult participants and from the parents or legal guardians of all pediatric participants.

### 2.2 Participant recruitment and clinical characterization

A total of 21 patients with angiographically confirmed Moyamoya disease (MMD) were prospectively enrolled from the Department of Neurosurgery at Xuanwu Hospital between 2019 and 2022. MMD shows a female predominance in several East Asian and Western cohorts, although the magnitude of sex differences varies among populations [28]. The cohort included 10 adult patients (Age: 38.70 ± 12.22 years; 70% for female) and 11 pediatric patients (Age: 8.55 ± 2.16 18 years; 54.5% for female). The diagnosis was established in accordance with the latest Japanese guidelines, based on clinical presentation, magnetic resonance imaging (MRI), and digital subtraction angiography (DSA) findings. Patients with moyamoya syndrome secondary to known etiologies (e.g., atherosclerosis, autoimmune disease, cranial irradiation) were excluded. Ten age- and sex-matched healthy adult volunteers with no history of cerebrovascular or severe systemic disease were recruited as the control group (HCs; Age: 41.00 ± 14.98 years; 70% for female). Demographic information, clinical history (including hypertension, smoking, and type of onset), and baseline laboratory data (lipid profile, glucose) were meticulously collected for all participants (Table 1, Table 2). Disease severity was graded using the Suzuki staging system based on DSA. Adult MMD patients had significantly lower serum LDL and total cholesterol levels than HCs, whereas other parameters, including age and sex, were comparable between groups.

**Table 1:** The Demographic and clinical parameters of healthy control and adult MMD patients.

Baseline characteristics	Control group (n = 10)	Adult MMD group (n = 10)	t	P-value
**General Information**				
Age(yrs)	41.00 ± 14.98	38.70 ± 12.22	1.11	0.297
Female	7 (70)	7(70)	-	0.686
**History disease**				
Hypertension	1(10)	2(20)		0.500
Smoking	2(20)	3(30)		0.500
Alcohol	1(10)	0(0)		0.500
**Experimental test:**				
LDL (mmol/L)	2.42 ± 0.70	1.61 ± 0.54	3.24	0.010
HDL (mmol/L)	1.01 ± 0.27	1.03 ± 0.38	−1.11	0.915
TC (mmol/L)	4.14 ± 0.89	3.02 ± 0.69	3.97	0.016
Glu (mmol/L)	5.36 ± 1.54	6.56 ± 3.33	−1.77	0.111
TG (mmol/L)	1.37 ± 1.09	1.03 ± 0.49	1.24	0.245

**Table 2:** The Demographic and clinical parameters of adult MMD and pediatric MMD patients.

Baseline characteristics	Pediatric MMD group (n = 11)	Adult MMD group (n = 10)	*t*	P-value
**General Information**				
Age(yrs)	8.55 ± 2.16	38.70 ± 12.22	−7.69	<0.001
Female	6 (54.5)	7(70)	-	0.392
Age at onset	7.77 ± 2.05	36.90 ± 12.10	−7.52	<0.001
**Type of onset**				
Infarction	3(27.3)	7(70)	-	0.063
TIA	7(63.6)	2(20)	-	0.056
Epilepsy	2 (18.2)	0 (0)	-	0.262
**Experimental test:**				
LDL(mmol/L)	2.06 ± 0.41	1.61 ± 0.54	2.18	0.042
HDL(mmol/L)	1.34 ± 0.39	1.03 ± 0.38	1.84	0.082
TC(mmol/L)	3.95 ± 0.47	3.02 ± 0.69	3.64	0.151
GLu(mmol/L)	4.96 ± 0.57	6.56 ± 3.33	−1.57	0.132
TG(mmol/L)	1.36 ± 0.88	1.03 ± 0.49	1.08	0.296

### 2.3 Blood sample collection and serum preparation

Peripheral venous blood (10 mL) was drawn from each participant using standard venipuncture techniques and collected in serum separation tubes. Samples were allowed to clot at room temperature for 30 min and then centrifuged at 3000 × g for 15 min at 4°C to isolate serum. The supernatant (serum) was carefully aliquoted into RNase/DNase-free tubes, flash-frozen in liquid nitrogen, and stored at −80°C until further processing to preserve exosomal integrity.

### 2.4 Small extracellular vesicle isolation, purification, and characterization

Serum-derived EVs were isolated from 1 mL of thawed serum per sample using the exoRNeasy Midi Kit (Qiagen, Cat# 77144) according to the manufacturer's protocol, which combines membrane affinity binding and column purification. For Transmission Electron Microscopy (TEM), 10 μL of purified EV suspension was applied to a carbon-coated copper grid, negatively stained with 2% phosphotungstic acid for 90 s, and air-dried. Images were captured using a Tecnai G2 Spirit TEM (Thermo Fisher Scientific) operated at 120 kV. Nanoparticle Tracking Analysis (NTA) was performed to determine particle size distribution and concentration using a NanoSight NS500 system (Malvern Panalytical) equipped with a 405 nm laser. Samples were appropriately diluted in sterile phosphate-buffered saline (PBS) to achieve optimal particle counting. Three 60-s videos were recorded for each sample and analyzed using NTA software (Version 3.0). Dynamic Light Scattering (DLS) for hydrodynamic diameter and zeta potential measurement was conducted using a Zetasizer Nano ZS90 (Malvern Panalytical). For immunoblotting, exosomal proteins were lysed in RIPA buffer. Protein concentration was quantified using the RC DC Protein Assay (Bio-Rad). SDS-PAGE separated equal amounts of protein, transferred to PVDF membranes, and probed with the following primary antibodies overnight at 4°C: anti-CD9 (Proteintech, 60232-1, 1:1000), anti-TSG101 (Absin, abs115706, 1:1000), anti-CD63 (Santa Cruz, sc5275, 1:200), and anti-Calnexin (Proteintech, 10427-2, 1:500). HRP-conjugated secondary antibodies (Beyotime) and enhanced chemiluminescence were used for detection.

### 2.5 Exosomal RNA extraction and small RNA sequencing

Total RNA, including small RNAs, was extracted from the purified EV fraction using the exoRNeasy Midi Kit according to the manufacturer's protocol. RNA for small RNA sequencing was purified from the EV-enriched fraction using the RNA purification procedure of the same kit. Separate EV-enriched aliquots were processed for protein extraction and Western blot analysis. RNA concentration and purity were assessed using a NanoDrop 2000 spectrophotometer (Thermo Scientific). RNA integrity was verified by agarose gel electrophoresis. Small RNA libraries were sequenced on an Illumina NextSeq 500 platform using single-end reads. Briefly, after 3′ and 5′ adaptor ligation and cDNA synthesis, libraries were amplified by PCR and size-selected for fragments of 140-160 bp.

### 2.6 Bioinformatic analysis of sequencing data

Raw sequencing reads were processed using an in-house pipeline. Adapter sequences and low-quality bases were trimmed using cutadapt (v3.4). Clean reads were aligned to the human reference genome (GRCh38) using Bowtie2 (v2.4.2). Small RNA annotation was performed by mapping to miRBase (v22) for miRNAs and to GENCODE (v35) for mRNAs and lncRNAs. Read counts for each RNA species were generated using featureCounts. Differential expression analysis was analyzed using DESeq2 (v1.36.0) in R, with significance defined as ∣log2 fold change∣ > 1 and adjusted P-value <0.05.

### 2.7 Weighted Gene Co-expression Network Analysis (WGCNA)

WGCNA was performed on the normalized miRNA expression matrix (variance-stabilized counts) using the WGCNA R package (v1.71). A soft-thresholding power (β = 6) was selected to achieve a scale-free topology (scale-free R^2^ > 0.85). Because of the small sample size, WGCNA was used only as an exploratory module-detection approach. A signed topological overlap matrix (TOM) was constructed, and modules were identified using hierarchical clustering with a minimum module size of 30. Module eigengenes (MEs) were calculated, and their correlations with clinical traits (disease status: MMD = 1, HC = 0; Suzuki score) were assessed. Modules with a correlation P-value <0.05 were considered significant. Functional enrichment analysis for the predicted target genes of miRNAs within significant modules was performed using the clusterProfiler package (v4.4.4) based on the Gene Ontology (GO) database.

### 2.8 Construction of the competing endogenous RNA (ceRNA) Network

Differentially expressed miRNAs, mRNAs, and lncRNAs were used for network construction. Experimentally validated and predicted miRNA-mRNA interactions were retrieved using the multiMiR R package (v1.20.0), integrating data from miRTarBase, TarBase, and TargetScan. miRNA-lncRNA interactions were obtained from the ENCORI (starBase) database. To build the ceRNA network, we first identified miRNA-mRNA and miRNA-lncRNA pairs with opposite expression trends (negative correlations) and significant correlations (Spearman's ∣ρ∣ > 0.6, P < 0.05). These pairs were then integrated to form lncRNA-miRNA-mRNA regulatory axes. The final network was visualized and analyzed using Gephi software (v0.10.1) with the ForceAtlas2 layout. Nodes with a high degree of connectivity were identified as network hubs.

### 2.9 Machine learning model development and evaluation

To develop a diagnostic classifier, we used the randomForest R package (v4.7-1.1). The dataset, comprising the expression levels of the top differentially expressed miRNAs, was randomly split into a training set (80% of samples) and an independent test set (20%), stratified by disease status. The model was trained on the training set using 10-fold cross-validation to tune the hyperparameter mtry (number of variables randomly sampled as candidates at each split). The mean decrease in Gini impurity is a measure of feature importance. The model's performance was evaluated on the held-out test set using the Receiver Operating Characteristic (ROC) curve, and the Area Under the Curve (AUC) was calculated with the pROC package (v1.18.0). The data-splitting and model-training process was repeated 50 times to assess stability, and the average performance metrics are reported. To reduce data leakage, feature selection was conducted within each training fold. Model performance was evaluated using repeated stratified cross-validation. Permutation testing was used to assess whether model performance exceeded chance expectation.

### 2.10 Statistical analysis

Continuous variables are presented as mean ± standard deviation (SD) or median with interquartile range (IQR) as appropriate. Differences between the two groups were assessed using the two-tailed Student's t-test or the Mann-Whitney *U* test. Comparisons among three or more groups were performed using one-way ANOVA or the Kruskal-Wallis test. Categorical variables are presented as frequencies and percentages and were compared using the Chi-square test or Fisher's exact test. All statistical analyses were performed using R (v4.2.2) or GraphPad Prism (v9.0). A P-value <0.05 was considered statistically significant.

## 3 Results

### 3.1 Characterization of serum EVs and exosomal RNA landscape

**Fig. 1:**
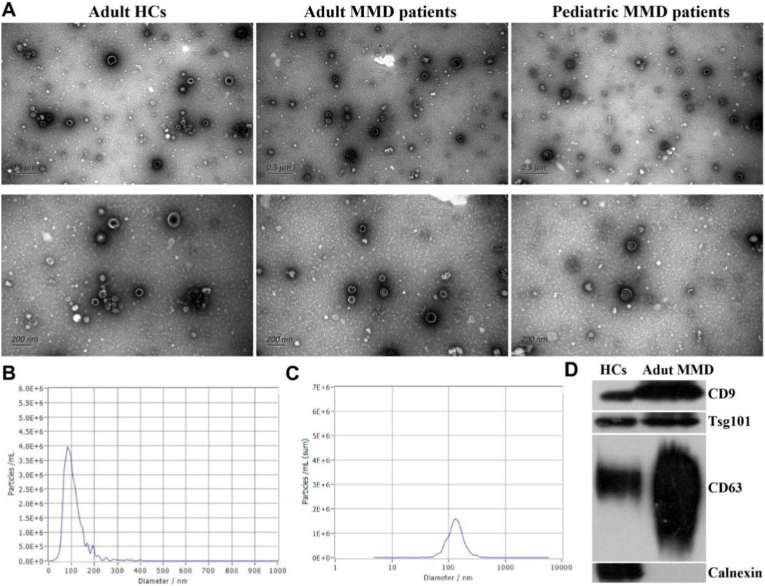
Isolation and characterization of serum-derived EVs. (A) Representative transmission electron microscopy (TEM) images of EVs from a healthy control (HC), an adult MMD patient, and a pediatric MMD patient. Scale bars: 100 nm; insets show higher-magnification views of bilayer morphology (arrows). (B) Nanoparticle tracking analysis (NTA) size distribution of EVs from an adult MMD patient (mean size = 128 nm; mode = 115 nm). (C) Dynamic light scattering (DLS) hydrodynamic diameter distribution (polydispersity index = 0.18) and zeta potential (−28 mV). (D) Immunoblot analysis showing enrichment of exosomal markers CD9, TSG101, and CD63 in EV-enriched particles, with absence of the endoplasmic reticulum marker calnexin; Sup, depleted serum supernatant.

We successfully isolated serum EVs from all 31 samples (21 MMD patients, 10 controls). Transmission electron microscopy (TEM) confirmed cup-shaped bilayer vesicles (60-100 nm) (Fig. 1A), consistent with exosomal morphology. Nanoparticle Tracking Analysis (NTA) and Dynamic Light Scattering (DLS) showed a monodisperse population with a mean hydrodynamic diameter of ∼130 nm (Fig. 1B–C), matching established EV size ranges and immunoblotting validated enrichment of exosomal markers (CD9, TSG101, CD63) [29] and absence of the endoplasmic reticulum marker Calnexin (Fig. 1D and S1), confirming EV purity.

### 3.2 Differential exosomal miRNA signatures in MMD patients

**Fig. 2:**
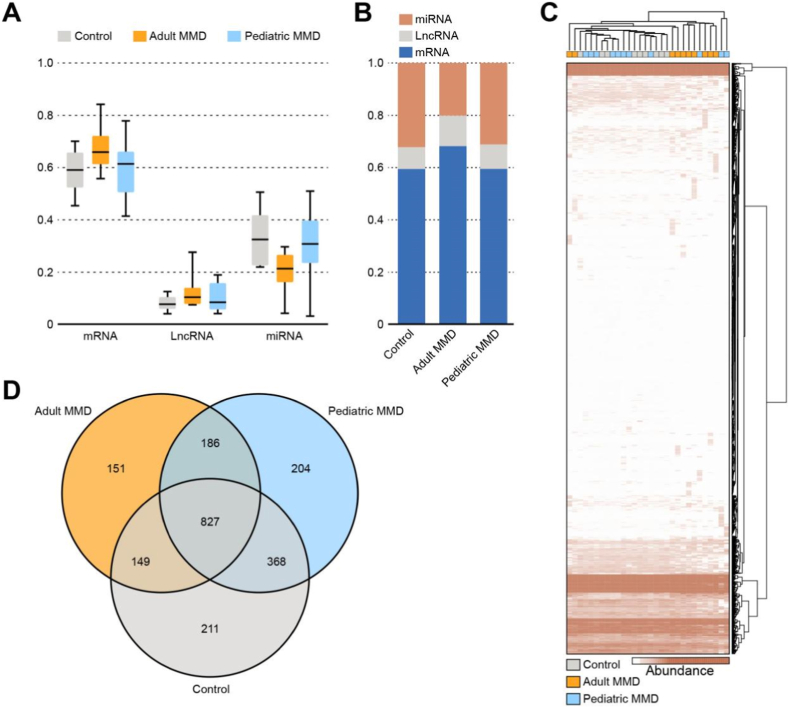
Profiling of exosomal RNA species and identification of differential miRNA signatures. (A) Box plot of RNA biotype distribution (miRNA, mRNA, lncRNA, other) across groups (center line, median; box, interquartile range; whiskers, 1.5× IQR). (B) Bar graph of average RNA biotype percentages per group. (C) Unsupervised hierarchical clustering heatmap of differentially expressed miRNAs (rows) across samples (columns). Sample dendrogram shows clear separation between adult controls and adult MMD patients. (D) Venn diagram depicting unique and shared miRNAs among groups.

Small RNA sequencing generated 12.8 ± 2.3 million clean reads per sample. Among mapped reads, mRNA-derived fragments represented the largest annotated fraction (62.3 ± 5.1%), whereas miRNAs constituted the major regulatory small RNA class analyzed for biomarker discovery (18.7 ± 3.2%), followed by lncRNAs (9.5 ± 2.4%) and other RNAs (9.5 ± 1.8%) (Fig. 2A–B). Unsupervised hierarchical clustering revealed distinct miRNA expression patterns: adult MMD patients clustered separately from controls, while pediatric MMD samples showed partial overlap but maintained a unique signature (Fig. 2C). We identified 132 differentially expressed (DE) miRNAs (|log2 fold change| > 1, adjusted P < 0.05) between MMD patients and controls, 78 upregulated and 54 downregulated. Among these, 63 DE miRNAs were specific to adult MMD, 41 to pediatric MMD, and 28 were shared between the two. Adult MMD patients exhibited significantly lower serum LDL and total cholesterol levels (Table 1), consistent with previous reports of dysregulation of lipid metabolism in MMD. Pediatric MMD patients had a higher incidence of transient ischemic attacks (TIAs) (63.6% *vs*. 20% in adults) and lower Suzuki staging scores (Table 2), reflecting age-related clinical heterogeneity. Venn diagram analysis identified 151 miRNAs uniquely expressed in adult MMD, 204 in pediatric MMD, and 186 shared between MMD groups (Fig. 2D), highlighting age-specific and disease-shared signatures. Taken together, exosomal sRNA sequencing identified the specific miRNA fingerprints and revealed the potential roles of miRNAs as biomarkers in MMD.

### 3.3 WGCNA identifies disease-associated miRNA modules

**Fig. 3:**
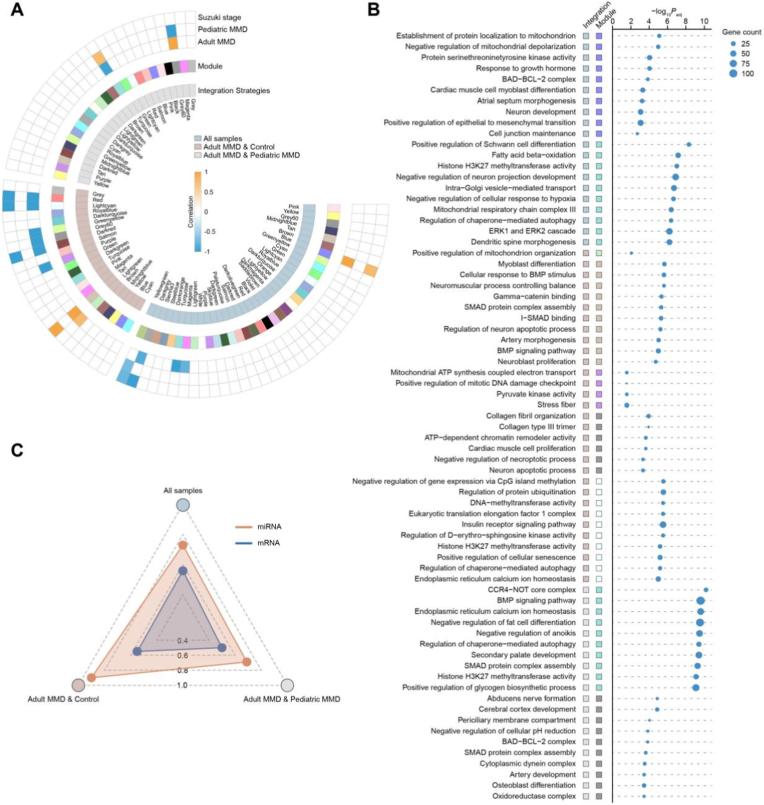
Weighted gene co-expression network analysis (WGCNA) identifies miRNA modules associated with MMD clinical traits. (A) Heatmap of module–trait correlations. Rows represent modules, columns represent clinical traits; color intensity indicates correlation coefficient; *P* < 0.05. (B) Gene Ontology (GO) biological process enrichment analysis of the “blue” and “lightcyan” modules (-log10(adjusted P)). (C) Radar chart comparing miRNA-versus mRNA-based module correlations with MMD traits across different integration strategies.

WGCNA was performed on normalized miRNA expression data (β = 6, scale-free R^2^ > 0.85) to identify co-expression modules[30]. We detected 12 modules, of which 4 showed significant correlations with clinical traits (P < 0.05) (Fig. 3A). The "blue module" (n = 42 miRNAs) was strongly positively correlated with adult MMD status (r = 0.73, P = 0.002) and Suzuki score (r = 0.68, P = 0.005). Gene Ontology (GO) enrichment analysis of its target genes highlighted pathways related to "mitochondrial membrane potential" (adjusted P = 3.2 × 10^−4^) and "regulation of reactive oxygen species metabolic process" (adjusted P = 5.7 × 10^−4^) (Fig. 3B). The "lightcyan module" (n = 35 miRNAs) was negatively correlated with MMD status (r = −0.65, P = 0.008) and enriched for "chromatin organization" (adjusted P = 2.1 × 10^−3^) and "histone modification" (adjusted P = 4.8 × 10^−3^), implicating epigenetic dysregulation. Radar chart analysis showed that miRNA-based modules had stronger correlations with MMD traits than mRNA-based modules across all integration strategies (Fig. 3C), confirming that miRNAs are superior biomarkers.

### 3.4 ceRNA Network reveals hub miRNAs

**Fig. 4:**
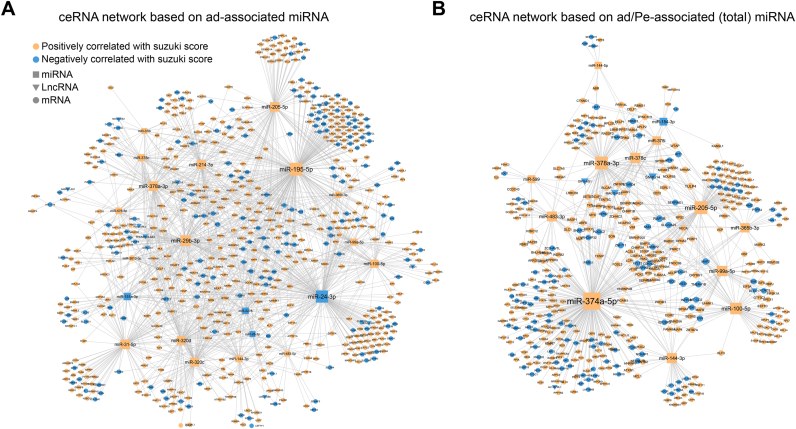
Competing endogenous RNA (ceRNA) network in MMD. (A) Global ceRNA network for adult MMD. Squares, miRNAs; circles, mRNAs; triangles, lncRNAs. Edges represent regulatory interactions; node size reflects degree centrality. (B) Focused subnetwork highlighting miR-205-5p and miR-374a-5p (red squares) and their target RNAs.

A putative ceRNA network was constructed using DE miRNAs, mRNAs, and lncRNAs (Spearman's |ρ| > 0.6, P < 0.05) to predict interactions and correlation filtering [31,32]. The adult MMD network included 12 miRNAs, 87 mRNAs, and 23 lncRNAs (Fig. 4A), with miR-205-5p and miR-374a-5p identified as central hubs (degree = 18 and 15, respectively) (Fig. 4B). These miRNAs targeted genes involved in vascular biology, including endothelial cell proliferation (e.g., VEGFA) and inflammation (e.g., TNFα). miR-205-5p and miR-374a-5p were consistently upregulated in adult (log2 FC = 2.1 and 1.8, respectively) and pediatric (log2 FC = 1.5 and 1.3, respectively) MMD patients, confirming their disease relevance.

### 3.5 Machine learning model for MMD diagnosis

**Fig. 5:**
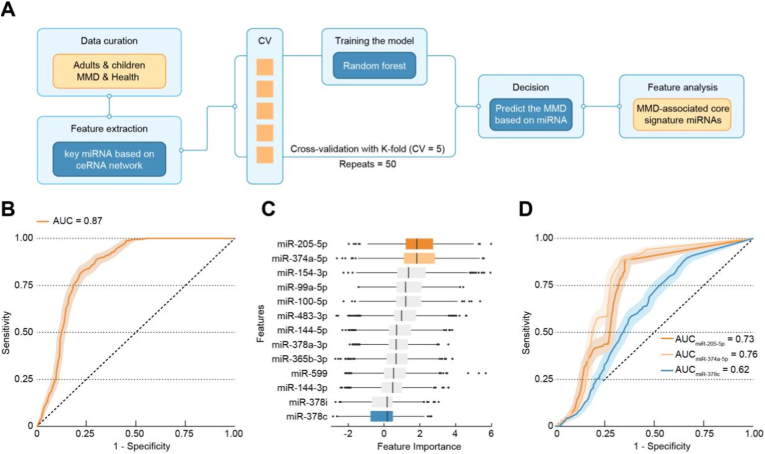
Machine learning model for MMD diagnosis based on exosomal miRNA profiles. (A) Analytical workflow: normalized miRNA expression data were split into training (80%) and test (20%) sets; feature selection and Random Forest classifier training were performed with 10-fold cross-validation. (B) Receiver operating characteristic (ROC) curve of the Random Forest model (AUC = 0.87, 95% CI: 0.78-0.96). (C) Feature importance of the top 15 miRNAs (mean decrease in Gini impurity). (D) Comparative ROC curves: single-miRNA (miR-205-5p, AUC = 0.72) versus multi-miRNA model (AUC = 0.87).

A Random Forest classifier was trained on the top 50 DE miRNAs (80% training set, 20% test set, 10-fold cross-validation)[33]. The model achieved an AUC of 0.87 (95% CI: 0.78–0.96), with sensitivity = 0.83 and specificity = 0.80 (Fig. 5A–B). Feature importance analysis ranked miR-205-5p and miR-374a-5p as the top two contributors (Fig. 5C). A single-miRNA model using miR-205-5p had an AUC of 0.72 (95% CI: 0.60–0.84), while the multi-miRNA signature showed superior performance (Fig. 5D), highlighting the value of combinatorial biomarkers. Model stability was confirmed by 50 iterations (mean AUC = 0.85 ± 0.03).

## 4 Discussion

This study establishes an integrative liquid biopsy platform for MMD diagnosis by combining exosomal miRNA profiling, systems biology, and machine learning. Serum EVs from MMD patients carry distinct miRNA fingerprints; WGCNA reveals miRNA modules linked to mitochondrial function and epigenetic regulation as disease-associated; miR-205-5p and miR-374a-5p function as central regulatory hubs and diagnostic biomarkers; and a Random Forest model incorporating these signatures achieves diagnostic performance (AUC = 0.87). However, these findings require technical validation, independent cohort validation, and evaluation against disease-control populations before clinical diagnostic application.

The application of WGCNA shifted our analysis from a list of differentially expressed miRNAs to the discovery of functionally coherent modules[34]. The strong association of the "blue" module, enriched for miRNAs targeting mitochondrial and oxidative stress pathways, with adult MMD is particularly compelling. Mitochondrial dysfunction and oxidative damage are increasingly recognized as central mediators of endothelial cell injury, blood-brain barrier disruption, and vascular remodeling in cerebral small vessel disease and stroke[35,36]. Our data suggest these mechanisms may be prominently encoded in the exosomal miRNA signature of MMD, offering a new molecular perspective on its pathogenesis. Similarly, the link between the "lightcyan" module and epigenetic regulation opens a novel avenue for investigation, as epigenetic modifications are key regulators of gene expression in response to ischemia and inflammation[37].

The ceRNA network analysis provided a putative regulatory framework[38], with miR-205-5p and miR-374a-5p emerging as central hubs. While the functional role of these specific miRNAs in cerebrovascular disease requires experimental validation, their known biology offers plausible connections. miR-205-5p has been implicated in regulating epithelial-mesenchymal transition, cell proliferation, and angiogenesis in cancer[39], processes analogous to the pathological vascular remodeling in MMD. miR-374a-5p is involved in inflammatory responses and endothelial cell function[40]. Adult MMD and adult healthy-control samples were used for the primary differential-expression and exploratory classification analyses. Pediatric MMD samples were analyzed descriptively because age-matched pediatric controls were not available. Their consistent identification as top features in our machine learning model, which achieved an AUC of 0.87, strongly supports their utility as biomarkers. The superior performance of the multi-feature model over single miRNAs highlights the complex, multifactorial nature of MMD and the power of combinatorial biomarker panels.

## 5 Clinical and translational implications

The diagnostic model developed here represents a significant step toward a blood-based, non-invasive test for MMD. Such a tool could be an earlier diagnosis in symptomatic individuals, facilitate screening of at-risk family members, and provide an objective means to monitor disease activity or response to revascularization. Moreover, the identified miRNA hubs and their associated pathways, particularly those involving mitochondrial function and epigenetic regulation, suggest novel therapeutic targets that could complement surgical intervention.

## 6 Limitations and future directions

This study has several limitations that chart the course for future research. First, the sample size, while sufficient for this discovery-phase investigation, necessitates validation in larger, independent, and multi-ethnic cohorts to ensure generalizability. Meanwhile, the lack of pediatric healthy controls prevents separation of age-associated miRNA differences from disease-associated differences in children. Second, our cohort included only ischemic-onset MMD patients; future studies should incorporate hemorrhagic-onset cases to determine if the signature is phenotype-specific. Third, while our analytical pipeline suggests mechanistic links, direct experimental validation of the roles of miR-205-5p and miR-374a-5p, and their target networks, in relevant *in vitro* (e.g., endothelial cell cultures under hypoxia) and in vivo models is crucial to establish causality. The candidate miRNAs identified here, including miR-205-5p and miR-374a-5p, require validation by orthogonal methods such as RT-qPCR and in independent, age-matched cohorts before clinical application can be considered. Fourth, longitudinal sample collection will be essential to determine if these miRNA signatures can track disease progression, predict stroke risk, or monitor response to revascularization therapy. However, Serum small EV-associated miRNAs may reflect systemic vascular, immune, endothelial, and platelet-related processes associated with MMD, but they are not brain- or vessel-specific.

## 7 Conclusion

In summary, this exploratory study identifies candidate serum small EV-associated miRNAs that may distinguish adult MMD patients from healthy controls in a small discovery cohort. We establish a diagnostic paradigm for MMD by integrating serum exosomal miRNA profiling with advanced computational biology. Moving beyond correlative observations, we provide a systems-level view in which coherent miRNA modules link to key pathological processes, with miR-205-5p and miR-374a-5p emerging as central regulators within a putative ceRNA network. The translation of this molecular signature into a high-performance machine learning classifier (AUC = 0.87) underscores its immediate potential as a non-invasive diagnostic tool. Moreover, our findings implicate mitochondrial and epigenetic pathways in MMD pathogenesis, opening new avenues for mechanistic investigation and therapeutic development.

## Ethics approval

This study was conducted in accordance with the World Medical Association Declaration of Helsinki (Ethical Principles for Medical Research Involving Human Subjects). It was approved by the Ethical Review Committee of Xuannwu Hospital of Capital Medical University (2023[051]). Informed consents were obtained from all participants. All patients provided written informed consent to use their biological specimens for research purposes.

## Funding

This work was supported by the Noncommunicable Chronic Diseases-National Science and Technology Major Project (2024ZD0539600), Xuanwu Hospital Science Program for Fostering Young Scholars (QNPY2021031), the National Natural Science Foundation of China (32171370), the Beijing Natural Science Foundation (L248075 and F262038), and the National Basic Research Plan of China (2025YFA1804601).

## CRediT authorship contribution statement

**Lin Yan:** Data curation, Formal analysis, Funding acquisition, Investigation, Methodology, Resources, Software, Validation, Visualization, Writing – original draft. **Hao Ding:** Data curation, Formal analysis, Methodology, Resources, Software, Validation. **Hanqing Chen:** Conceptualization, Funding acquisition, Project administration, Supervision, Writing – review & editing.

## Declaration of competing interest

The authors declare no competing interests.

## Data Availability

Data will be made available on request.
