# Supplementary material for: Exploratory profiling of serum small extracellular vesicle-associated miRNAs as candidate biomarkers for Moyamoya disease
Source: Biochem Biophys Rep. 2026 Jul 20;47:102686. doi: 10.1016/j.bbrep.2026.102686 (PMC13393599; doi:10.1016/j.bbrep.2026.102686)
Supplement: Multimedia component 1 [file mmc1.docx]

**Supplementary material**

**Exploratory profiling of serum small extracellular vesicle-associated miRNAs as candidate biomarkers for Moyamoya disease**

Lin Yan ^1,2,#^, Hao Ding ^3,#^, Ruifang Zhao ^3^, Hanqing Chen ^4,^*

^1^ Department of Neurosurgery, Xuanwu Hospital, Capital Medical University, Beijing 100053, China

^2^ China International Neuroscience Institute (China-INI), Beijing 100053, China

^3^ Beijing Key Laboratory for Drug Delivery Nanocarriers, CAS Center for Excellence in Nanoscience, National Center for Nanoscience and Technology, Beijing 100190, China

^4^ Beijing Key Laboratory of Environment and Aging, Department of Nutrition & Food Hygiene, School of Public Health, Capital Medical University, Beijing 100069, China

^#^ These authors contributed equally to this work

* Corresponding authors:

1. mail address: chenhq@ccmu.edu.cn (H. Chen).


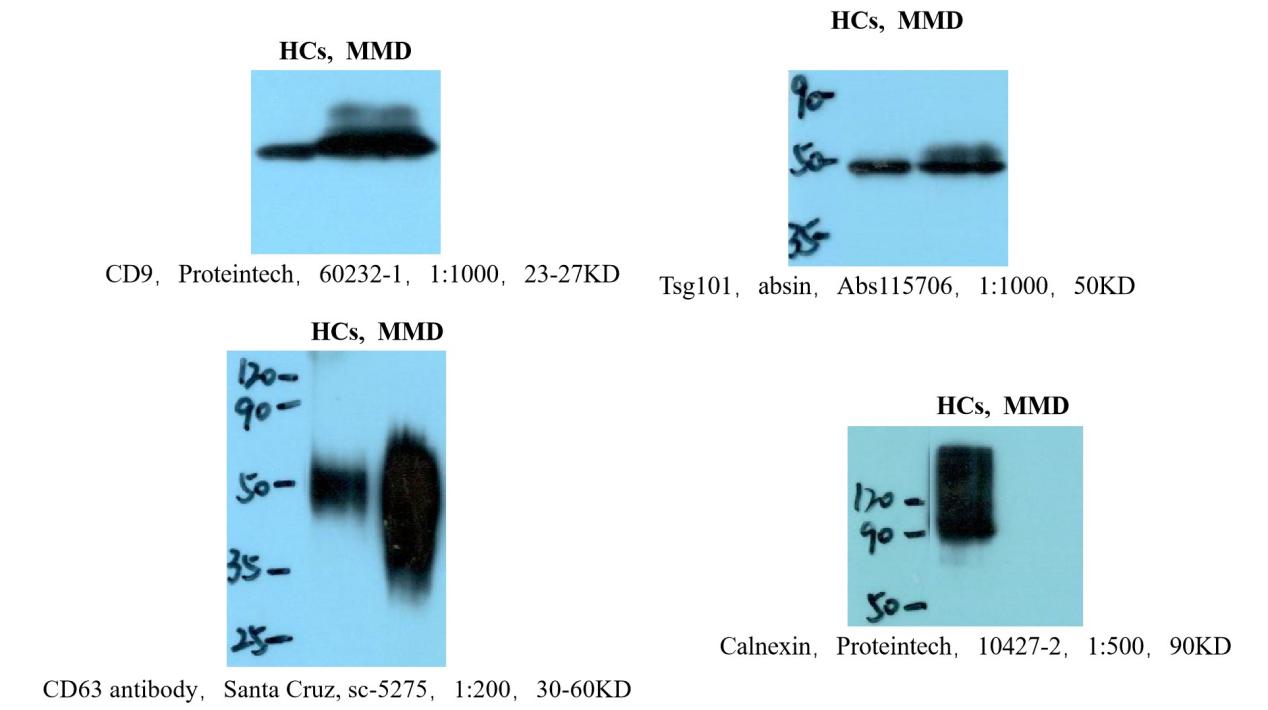


**Figure S1.** The full uncropped Gels and Blots image(s) used in Figure 1D.
